# Hysteroscopic myomectomy with the IBS® Intrauterine Bigatti Shaver: A Retrospective Comparative Analysis of the impact of rotational speed and aspiration flow rate

**DOI:** 10.52054/FVVO.15.1.063

**Published:** 2023-03-31

**Authors:** S Zhang, W Di, Y Wang, J Shi, X Yin, Y Zhang, A Zhao, R Campo, G Bigatti

**Affiliations:** Sino European Life Expert Centre (SELEC), Department of Gynaecology and Obstetrics, Renji Hospital, Shanghai Jiao Tong University School of Medicine, China; Shanghai Key Laboratory of Gynaecologic Oncology, Shanghai, China; Life Expert Centre, Leuven Belgium

**Keywords:** Intrauterine Bigatti Shaver, submucous myomas, hysteroscopic myomectomy, rotational speed, aspiration flow rate

## Abstract

**Background:**

Myoma removal remains a challenge hysteroscopically including for the “IBS®” Intrauterine Bigatti Shaver technique.

**Objective:**

To evaluate whether the Intrauterine IBS® instrument settings and the myoma size and type are prognostic factors for the complete removal of submucous myomas using this technology.

**Materials and methods:**

This study was conducted at the San Giuseppe University Teaching Hospital Milan, Italy; Ospedale Centrale di Bolzano - Azienda Ospedaliera del Sud Tirolo Bolzano, Italy (Group A) and the Sino European Life Expert Centre-Shanghai Jiao Tong University School of Medicine Affiliated Renji Hospital, Shanghai, China (Group B). In Group A: surgeries were performed between June 2009 and January 2018 on 107 women using an IBS device set to a rotational speed of 2,500 rpm and an aspiration flow rate of 250ml/min. In Group B: surgeries were performed between July 2019 and March 2021 on 84 women with the instrument setting to a rotational speed of 1,500 rpm and aspiration flow rate of 500 ml/min. Further subgroup analysis was performed based on fibroid size:<3 cm and 3-5 cm. Both Groups A and B were similar in terms of patient age, parity, symptoms, myoma type and size. Submucous myomas were classified according to the European Society for Gynaecological Endoscopy classification. All patients underwent a myomectomy with the IBS® under general anaesthesia. The conventional 22 Fr. Bipolar Resectoscope was used in cases requiring conversion to the resection technique. All surgeries were planned, performed and followed by the same surgeon in both institutions.

**Main outcome measures:**

Complete resection rates, total operation time, resection time and used fluid volume.

**Results:**

Complete resection with the IBS® Shaver was seen in 93/107 (86.91%) in Group A versus 83/84 (98.8 %) in Group B (P=0.0021). Five patients (5.8%) in Subgroup A1 (<3 cm) and nine patients (42.9%) in Subgroup A2 (3cm~5cm) could not be finished with the IBS (P<0.001, RR=2.439), while in Group B only one case (8.3%) in Subgroup B2 (3cm~5cm) underwent a conversion to bipolar resectoscope (Group A: 14/107=13.08% vs. Group B: 1/84=1.19%, P=0.0024). For <3cm myomas (subgroup A1 versus B1) there was a statistically significant difference in terms of resection time (7.75±6.363 vs. 17.28±12.19, P<0.001), operation time (17.81 ± 8.18 vs. 28.19 ±17.614, P<0.001) and total amount of fluid used (3365.63 ± 2212.319 ml vs. 5800.00 ± 8422.878 ml, P<0.05) in favour of Subgroup B1. For larger myomas, a statistical difference was only observed for the total operative time (51.00±14.298 min vs. 30.50±12.122 min, P=0.003).

**Conclusion:**

For hysteroscopic myomectomy using the IBS®, 1,500rpm rotational speed and 500ml/min aspiration flow rate are recommended as these settings result in more complete resections compared to the conventional settings. In addition, these settings are associated with a reduction in total operating time.

**What is new?:**

Reducing the rotational speed rate from 2500 rpm to 1500 rpm and increasing the aspiration flow rate from 250 ml/min to 500 ml/min improve complete resection rates and reduce operating times.

## Introduction

Uterine myomas are benign tumours of the female genital tract, and submucous myomas account for 5.5% to 16.6% of all uterine fibroids (Di Spiezio Sardo et al., 2008). Heavy or prolonged menstrual bleeding with anaemia, abnormal uterine bleeding, pelvic pain, infertility, and/or recurrent pregnancy loss are the symptoms frequently associated with the presence of submucous myomas (Emanuel, 2015). Several medical and surgical therapies are now available for the treatment of submucous myomas. The best treatment choice depends on the patient’s personal objectives and the efficacy of each therapeutic option. Although new alternative medical treatments are now available (Oxley et al., 2020), the double-flow bipolar resectoscope remains the gold-standard surgical device to approach submucous myomas. In 1976, Neuwirth and Amin described the first hysteroscopic excision of a submucous myoma (Neuwirth and Amin, 1976). Since then, mono- or bipolar resectoscopy has become a common procedure for the treatment of submucous myomas (Di Spiezio Sardo et al., 2008). Unfortunately, many technically related problems, such as fluid overload, uterine perforation, impaired visual field, and long learning curve remain unresolved (Pasini and Belloni, 2001). With the advance of new hysteroscopic instruments, a new approach to tissue removal was introduced. Currently, there are three main tissue removal systems: Truclear®, Myosure® and Intrauterine Bigatti Shaver (IBS®). Most studies have reported the benefits of hysteroscopic tissue removal systems such as shorter operative time, higher total resection rate, and higher patient acceptability (Georgiou et al., 2018; Meulenbroeks et al., 2017; Yin et al., 2018). Myoma size and consistency still limit the use of tissue removal systems for this indication which accounts for 10-15% of all operative hysteroscopies. The IBS® was studied to improve on the results of conventional bipolar resectoscopy. In June 2009, the first myomectomy with the use of the IBS® was performed (Bigatti, 2010). And in 2014, Bigatti and colleagues compared, for the first time, the IBS® with the conventional bipolar resectoscope (Bigatti et al., 2014). The IBS® was able to remove in a single-step procedure 93.5% of submucous myomas smaller than 3 cm in diameter, 62.5% of which were type II. The IBS®, by removing the tissue chips at the same time as resection, has addressed several issues that pertained to conventional resectoscopy (Bigatti et al., 2012; Jansen et al., 2000; Witz et al., 1993). Currently, almost 95% of major operative hysteroscopic procedures, such as polypectomies and myomectomies for submucous myomas smaller than 4 cm can be successfully performed in a single- step procedure by the IBS® (Bigatti et al., 2012a; Bigatti et al., 2014). This result confirms what was previously reported by Emanuel and Wamsteker with a similar device (Emanuel and Wamsteker, 2005). Over the last 10 years, there have been further improvements in the IBS® technical features. Generally, a power of up to 5,000 oscillating rotation power per minute and a 200 to 1,000 ml/ min aspiration flow rate are offered by the IBS®. Since the rotating and oscillating movements of the shaving system inner blade have important effects on tissue cutting, with the present study, we aimed to evaluate whether IBS® settings affect the success rate of hysteroscopic myomectomy.

## Materials and methods

### Study Design and Population

This was a retrospective analysis of data collected between January 2018 and March 2021 at the San Giuseppe University Teaching Hospital Milan, Italy and at Ospedale Centrale di Bolzano - AziendaOspedaliera del Sud Tirolo Bolzano, Italy (Group A) and from July 2019 to March 2021 at the Sino European Life Expert Centre - Shanghai Jiao Tong University Affiliated Renji Hospital Shanghai, China (Group B). The source population included women undergoing hysteroscopic submucous myomectomy with the IBS®. Diagnosis of submucous myomas was made in each patient by 2D ultrasound examination and confirmed by diagnostic hysteroscopy. Submucous myomas were classified according to the European Society for Gynaecological Endoscopy (ESGE) system (Wamsteker et al., 1993). Each group of patients was further divided into two subgroups according to the size of the submucous myomas. Subgroups A1 and B1 included patients with a diameter of submucous myomas smaller than 3 cm, and subgroups A2 and B2 included patients with a diameter of submucous myomas between 3 cm and 5 cm. All types of myomas (type 0, I and II), according to the ESGE classification, requiring a hysteroscopic myomectomy with the IBS® were included in the study. Patients with endometrial polyps, non-hysteroscopic associated surgical pathologies such as cervical lesions and all types of submucous myomas larger than 5cm were excluded from the study.

### Equipment

All submucous myomectomies have been performed with the IBS® (Karl Storz SE & Co. KG). A detailed description of the equipment is included in a recent publication by Bigatti et al. (2020). In summary, the IBS® Shaver system is made up of two distinct instrumental components: the optical part that allows visualisation inside the uterine cavity and the cutting device called the ‘Shaver’. The optical component consists of a 90° angulated 6°optics (Karl Storz SE & Co. KG) with a double flow sheath and an extra operative channel into which a rigid shaving system is introduced. The double-flow sheath is connected to a peristaltic pump (HamouEndomat ® Karl Storz SE & Co. KG) to maintain distension and visualisation inside the uterine cavity. The total diameter of the optical system is 24Fr (8mm). The rigid shaving system is made up of two reusable hollow metal tubes that fit into each other. The inner tube rotates within the outer tube and is connected both to a handheld (Drillcut-X® II Karl Storz SE & Co. KG) motor drive unit (Unidrive® S III Karl Storz SE & Co. KG) and to an extra roller pump (Endomat® LC Karl Storz SE & Co. KG) controlled by a foot pedal. In this study, we performed all myomectomies using the SB/shaver blade (SB Blade® Karl Storz SE & Co. KG) which has a diameter of 4 mm and a window surface of 25 mm2. Rotation and oscillation of the shaving system inner blade cut the tissue to allow specimen aspiration for histology. Intraoperative bleeding was managed with a bipolar coagulation electrode (SB Blade® Karl Storz SE & Co. KG) introduced through the straight operative channel of the optics. When there was a need for conversion from the IBS® resectoscopy, the 22 Fr conventional bipolar resectoscope (Karl Storz SE & Co. KG) with 4 mm loops was used.

### Surgical Procedure

**Figure 1:**
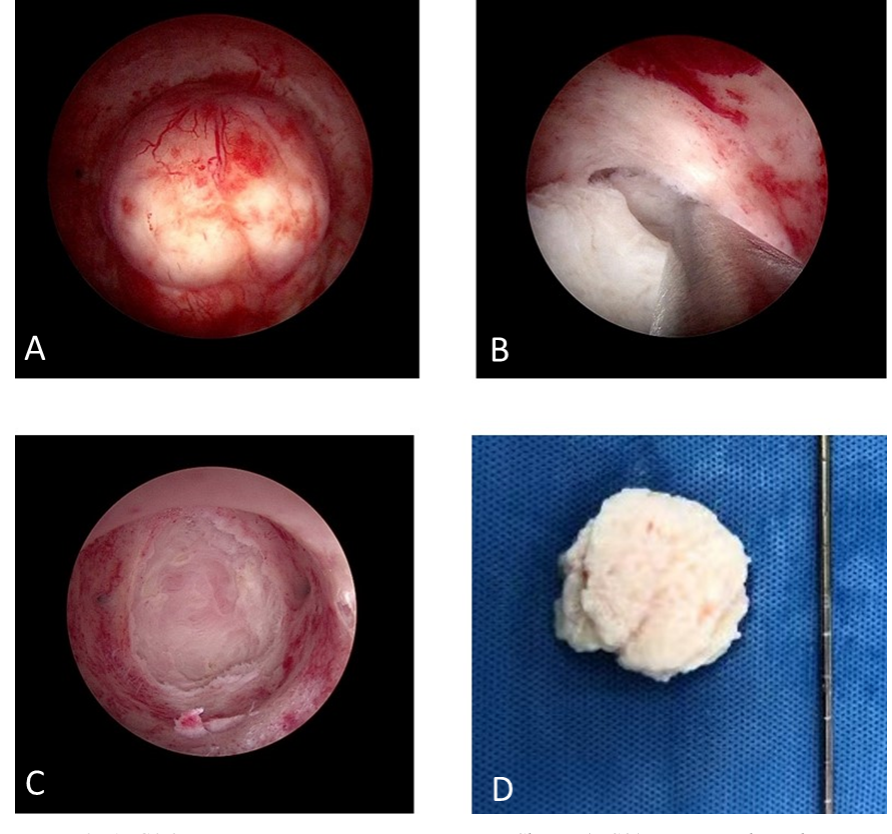
A: G1 3,5 cm myoma. B: Intrauterine Bigatti Shaver (IBS®) in action along the myoma capsule. C: Clear vision of the myoma fovea. D: Fragments of the myoma after complete removal.

All operations were performed by the same surgeon (GB) under general anaesthesia, and a standard gynaecological set up was used in all operating theatres. Upon dilatation of the cervical canal up to number 8.5 of Hegar, the Shaver optics was introduced into the uterine cavity. We used a normal isotonic saline solution for distension and irrigation. The maximum flow setting was 450 ml/min with an intrauterine pressure of less than 95 mmHg. Once the myoma was identified, the rigid shaving system was inserted inside the straight operative channel of the optics, and the procedure began. Aspiration started only when the first switch of the pedal was pressed, while the second switch of the pedal activated rotation and oscillation of the shaving system’s inner blade to cut the tissue. The resected tissue was aspirated directly into a glass bottle. In Group A, we used an IBS® oscillating rotational speed of 2,500 rpm with an aspiration flow rate of 250ml/min, whereas in Group B, an IBS® rotational speed of 1,500 rpm with an aspiration flow rate of 500ml/min was used. For type 0 myomas, we started cutting the myoma from its top until we reached the base. For type I and II myomas, we first located the cleavage plane between the capsule and the surrounding endometrium and then shaved and aspirated the myoma from its ‘fovea’. For all procedures we always used the SB/ 25 mm^2^ Blade (Figure 1). The procedure was considered successful when complete removal of the myoma with a clean fovea was achieved. A bipolar probe was used to stop bleeding by precisely coagulating the vessels under monitoring.

### Measurement methods

In all cases, the myoma size, type and position were assessed by vaginal ultrasound and/or confirmed by diagnostic hysteroscopy according to the ESGE classification (Wamsteker et al., 1993). All clinical data such as cervical dilatation time, resection time, total operation time, fluid deficit, bleeding, hospitalisation time and complications were evaluated. For total operation time, we considered the time of the whole procedure including cervical dilatation. The resection time was measured from the moment the active shaver tip was visible inside the uterine cavity until resection completion. Correct fluid balance was calculated by checking the fluid aspirated by the Hamou Endomat (Hamou Endomat® Karl Storz SE & Co. KG) and by the roller pump (Endomat® LC Karl Storz SE & Co. KG) connected to the shaving system, along with the fluid collected in a graduated plastic bag placed under the patient. The number of second- step procedures and conversions to a conventional bipolar resectoscope has also been evaluated. A two-step procedure was planned when either a 2000 mL fluid deficit or a procedure length of 60 minutes was recorded. Conversions from the IBS® to bipolar resectoscopy were due to two reasons. The first occurred when the myoma consistency, combined with its size, was preventing its fast resection with the IBS®; and the second when reaching the fluid deficit limit there was still a need for removal of a small portion of a very hard myoma.

### Statistical analyses

The data were presented as mean ± standard deviation (SD). Statistical analysis was performed using the SPSS software (version 17.0, SPSS Inc., Chicago, IL, USA) including unpaired Student’s t-test and the Yates corrected chi-square test. Differences between groups have been considered statistically significant at P<0.05.

### Ethical approval

The Renji Hospital institutional ethical committee approved this analysis and all patients provided informed consent before each surgical procedure.

## Results

### Clinical and demographic characteristics data

Both groups A and B were similar in terms of patient age, parity, and symptoms (P>0.05, Table I). No significant differences in terms of myoma mean diameter (22.69 ± 10.995 mm vs. 23.68 ± 9.249 mm, P>0.05) or myoma type (P=0.632) were found. In addition, no significant differences in the number and size of each myoma type (type 0:18.65±9.736 mm vs. 23.96±10.312 mm, P=0.06; type I: 27.50±9.952 mm vs. 24.02±8.894 mm, P=0.228; type II: 26.50±12.030 mmvs.27.50±5.000 mm, P=0.877) between the two groups were found.

**Table I:** Demographic characteristics.

	Group A (n=107)	Group B (n=84)
Age	48.77±9.85	45.51±8.77
Symptoms		
None	54	40
Menorrhagia	25	25
Pelvic pain	14	9
Infertility	14	10
Myomas classification		
Type 0	49(45.8%)	32 (38.1%)
Type 1	32 (29.9%)	47(56.0%)
Type 2	26 (24.3%)	5 (6.0%)
Myomas diameter	22.69±10.995	23.68±9.249

Group A: rotational speed 2,500 rpm and aspiration flow rate of 250 ml/min. Group B: rotational speed 1,500 rpm and aspiration flow rate of 500 ml/min. Myomas were classified according to the classification of European Society for Gynaecological Endoscopy (ESGE); G0: No intramural extension; G1: Intramural extension <50%; G2: Intramural extension ≥50%.

### Completeness of myoma resection

Complete resection with the IBS® Shaver was seen in 93/107 (86.91%) in Group A versus 83/84 (98.8 %) in Group B (P=0.002). When the data were analysed according to myoma size <3cm, complete resection with the IBS® Shaver was seen in 81/86 (94.19%) in subgroup A1 versus 72/72 (100 %) in subgroup B1 (P=0.06). For myomas between 3 and 5 cm in maximum diameter, complete resection with the IBS ® Shaver was achieved in 12/21 (57.14 %) in subgroup A2 versus 11/12 (91.67 %) in subgroup B2 (P= 0.06). No perforation or other post-operative complications were reported in either of the groups. Histopathological examination of all resected tissue confirmed benign leiomyoma tissue in all patients.

### Resection time and total operation time

Perioperative data in Group A and B were reported in Tables II and III. There was a statistically significant difference in the resection time (P<0.001), the total operation time (P<0.001) and the total amount of fluid used (P<0.01) between Group A and B. No statistically significant differences in terms of fluid deficit and cervical dilatation between the two groups were found (Table III).

**Table II:** Perioperative data.

	Group A (n.107)	Group B (n.84)	P value
Dilatation time (min)	1.39±1.043	1.15±0.453	0.077
Resection time (min)	18.30±14.370	9.12±8.102	0.000
Total time (min)	30.23±18.538	19.51±9.462	0.000
In (ml)	5800.94±6332.299	3807.23±2694.157	0.008
Out (ml)	4423.33±3674.516	3156.02±2105.371	0.006
Deficit (ml)	790.66±901.627	651.21±809.358	0.270

Group A: rotational speed 2,500 rpm and aspiration flow rate of 250 ml/min. Group B: rotational speed 1,500 rpm and aspiration flow rate of 500 ml/min.

**Table III:** Surgical time and fluid balance according to the types of myomas (Type 0-2).

	Myoma diameter (mm)	Resection time (min)	Operation time (min)	In (ml)	Out (ml)	Deficit (ml)
Type 0						
Group A(n=49)	18.65±9.736	15.72±12.856	27.30±15.953	4820.45±4211.147	3987.50±3783.3961	607.95±616.436
Group B(n=32)	23.96±10.312	6.56±5.480	16.81±9.016	3214.29±2734.514	2714.29±2259.419	500.00±536.104
P value	0.060	0.001	0.003	0.078	0.113	0.449
Type 1						
Group A(n=32)	27.50±9.952	21.36±15.938	32.01±19.338	8100.00±10025.080	5442.59±3779.483	886.21±768.451
Group B(n=47)	24.02±8.894	12.10±9.622	22.39±10.217	4756.10±2754.274	3901.22±2059.323	854.88±1018.934
P value	0.228	0.004	0.009	0.046	0.030	0.889
Type 2						
Group A(n=26)	26.50±12.030	20.75±13.104	33.83±18.235	5595.65±3507.327	4356.53±2998.648	1160.87±1419.343
Group B(n=5)	27.50±5.000	5.75±2.987	16.25±2.500	2150.00±810.350	1700.00±476.095	450.00±404.145
P value	0.877	0.034	0.070	0.066	0.094	0.336

Group A: rotational speed 2,500 rpm and aspiration flow rate of 250 ml/min. Group B: rotational speed 1,500 rpm and aspiration flow rate of 500 ml/min.

For each different type of myomas type 0-II, a statistically significant difference in the resection time and total operation time between Group A and B was present. No statistically significant differences in the fluid deficit and cervical dilatation between the two groups were found (Table II).

When patients were further divided into two subgroups according to the myoma diameter (subgroups A1 and B1: size < 3 cm, and subgroups A2 and B2: size between 3 cm and 5 cm), we found statistically significant differences in the resection time (P<0.001), the operation time (P<0.001) and the total amount of fluid used (P=0.03) between subgroups A1 and B1. No statistically significant differences in the fluid deficit or cervical dilatation between the two subgroups were found (Table IV). Comparisons between subgroups A2 and B2 showed statistically significant differences in the total operation time (P=0.003). No statistically significant differences were found between the two subgroups in terms of the resection time, the total amount of fluid used, the fluid deficit and the dilatation time (Table IV).

**Table IV:** Surgical time and fluid balance according to the size of myoma.

Subgroups	Number	Resection time (min)	Operation time (min)	In (ml)	Out (ml)	Deficit (ml)
<3cm						
Subgroup A1	86/107 = 80.37 %	17.28±12.19	28.19±17.614	5800.00±8422.878	3538.10±2452.595	763.10±895.045
Subgroup B1	72/84 = 85.71 %	7.75±6.363	17.81±8.186	3365.63±2212.319	2824.22±1804.382	541.41±513.512
P value	0.4409	0.000	0.000	0.030	0.088	0.109
3~5cm						
Subgroup A2	21/107=19.62 %	26.83±18.531	51.00±14.298	8200.00±4410.845	7111.11±4239.235	1500.00±705.534
Subgroup B2	12/84 =14.28%	18.20±11.951	30.50±12.122	7260.00±3186.499	5700.00±2002.776	1560.00±1729.611
P value	0.4409	0.232	0.003	0.592	0.358	0.920

Group A: rotational speed 2,500 rpm and aspiration flow rate of 250 ml/min. Group B: rotational speed 1,500 rpm and aspiration flow rate of 500 ml/min. Subgroups A1/B1 (<3 cm myomas); Subgroups Group A2/B2 (3~5 cm myomas).

## Discussion

Our present study was designed to compare the performance of the IBS® with two different rotational speed and aspiration flow rate settings for submucous myomectomy. In Group A, a rotational speed of 2,500 rpm and an aspiration flow rate of 250ml/min were used; while in Group B, a rotational speed of 1,500 rpm with an aspiration flow rate of 500ml/min was applied. A significantly higher complete resection rate with the IBS® Shaver was seen in Group B (83/84, 98.8%) compared to Group A (93/107, 86.91%) (P=0.0021). In Group A, 14 patients underwent a second step with conversion to a conventional bipolar resectoscope; while in Group B only one case in Subgroup B2 (3cm~5cm) underwent conversion to bipolar resectoscope (14/107, 13.08% vs. 1/84, 1.19%, P=0.0024). These significant differences indicate an evident improvement in the performance of the IBS® in Group B. The IBS® was able to effectively enucleate myomas from their fovea, including removal of the intramural site of insertion with a high degree of precision (Figure 1). In addition, all patients treated with the IBS® in Group B showed a statistically significant reduction in resection time, total operative time and fluid utilisation, especially for all myomas types (0-II) smaller than 3cm in diameter (subgroup B1). The IBS ® was shown to be a minimally invasive and less operator- dependent tool than the resctoscope (Bigatti, 2010; Bigatti et al., 2012a; Bigatti et al., 2012b; Bigatti et al., 2014). We think the reduction in the rotational blade speed afforded more time for the tissue to enter the window blade and be removed. Furthermore, the increased aspiration flow rate, with closer contact of the window blade to the tissue, allowed a much faster tissue resection. We also found that the IBS® efficacy was affected more by the myoma size than the myoma type. Emanuel (2015) reported that the diameter of an intrauterine pathology is strongly related to the operation time. Their findings are supported by our results: improved total resection time, surgical time and amount of fluid used for all types of myomas in Subgroup B1 (<3cm). However, in the case of larger myomas, we found a significant reduction in the total surgical time in Subgroup B2 (3cm~5cm). It has been shown that the learning curve for the use of the IBS® is extremely short, and therefore, experience related to the use of this new device has little impact on the success rate of the procedure (Bigatti et al., 2012b). As a general principle, myomectomy is still a challenging hysteroscopic operation, and therefore, should always be performed by or under the supervision of an experienced hysteroscopist. What really adds value to the present study is that all procedures were performed by the same surgeon reducing additional inter-observer variation.the case of larger myomas, we found a significant reduction in the total surgical time in Subgroup B2 (3cm~5cm). It has been shown that the learning curve for the use of the IBS® is extremely short, and therefore, experience related to the use of this new device has little impact on the success rate of the procedure (Bigatti et al., 2012b). As a general principle, myomectomy is still a challenging hysteroscopic operation, and therefore, should always be performed by or under the supervision of an experienced hysteroscopist. What really adds value to the present study is that all procedures were performed by the same surgeon reducing additional inter-observer variation.

In conclusion, our study confirms that a rotational speed of 1500 rpm and aspiration flow rate of 500 ml/min with IBS ® achieve better performance in the removal of intrauterine submucous myomas. The improved results indicate the need to promote further technical research on hysteroscopic tissue mechanical removal systems to improve the success rates in cases of removal of large submucous myomas. The IBS® is currently the only reusable device and therefore, is a unique instrument in its field. Further prospective research, ideally in the form of double-blind randomised control studies should be performed to describe advantages and possible drawbacks of this new class of devices.
